# Comparison in Outcomes at Two-Years of Age of Very Preterm Infants Born in 2000, 2005 and 2010

**DOI:** 10.1371/journal.pone.0114567

**Published:** 2015-02-06

**Authors:** Lénaïg Abily-Donval, Gaëlle Pinto-Cardoso, Alexandra Chadie, Anne-Marie Guerrot, Stéphanie Torre, Stéphane Rondeau, Stéphane Marret, on behalf of the Perinatal Network of Haute—Normandie

**Affiliations:** 1 Department of Neonatal Medicine, Rouen University Hospital, Rouen, Haute-Normandie, France; 2 Equipe Region-Institut National de la Santé et de la Recherche Médicale, ERI 28, Neovasc, Institute for Biomedical Research and Innovation, School of Medicine, Rouen University, Rouen, Haute-Normandie, France; Robert Debre Hospital, FRANCE

## Abstract

**Objective:**

To investigate alteration in 2-year neurological/behavioral outcomes of very preterm infants born in a French level three neonatal intensive care unit.

**Methods:**

We conducted a prospective, comparative study of very preterm infants born before 33 weeks’ gestation at 5-year intervals in 2000, 2005 and 2010 at Rouen University Hospital. Neonatal mortality/morbidities, ante- and neonatal treatments, and at age 2 years motor, cognitive and behavioral data were collected by standardized questionnaires.

**Results:**

We included 536 very preterm infants. Follow-up rates at two years old were 78% in 2000, 93% in 2005 and 92% in 2010 respectively. No difference in gestational age, birthweight, neonatal mortality/morbidities was observed except a decrease in low grade subependymal/intraventricular hemorrhages. Care modifications concerned use of antenatal magnesium sulfate, breast-feeding and post-natal corticosteroid therapy. Significant improvement in motor outcome and dramatic decrease in cerebral palsy rates (12% in 2000, 6% in 2005, 1% in 2010, *p*<0.001) were observed, as were improvements in feeding behavior. Although a non significant difference to better psychosocial behavior was reported, there was no difference in cognitive outcome.

**Conclusions:**

Improvement in neuromotor outcome and behavior was reported. This could be due to multiple modifications in care: including administration of magnesium sulfate to women at risk of preterm birth, increase in breast-feeding, decrease in low grade subependymal/intraventricular hemorrhages, and decrease in post-natal corticosteroid therapy, all of which require further investigation in other studies. Extended follow-up until school age is mandatory for better detection of cognitive, learning and behavioral disorders.

## Introduction

Incidence of very preterm birth (VPT) in France increased between 1998 and 2003 but stabilized in 2010 at 2% of births [1]. Despite advances in neonatal care medicine with a gradual increase in survival, neonatologists are aware of sparing these children from lifelong conditions such as cerebral palsy (CP), cognitive/behavioral and learning difficulties. The pathogenesis of brain lesions and neurodevelopmental disorders at the origin of subsequent handicaps is complex and several ante-, peri- and neonatal risk factors have been identified [2–3]. CP, the most common and severe developmental motor disorder [4–5] is reported in EPIPAGE 1 study in 8.2% at age 2 years of VPT infants born before 33 weeks’ gestation in nine regions of France [6]. In addition, cognitive, motor and behavioral impairments at age 8 years were found in 31%, 14% and 6% respectively [7]. These disabilities predict learning difficulties, which are very frequent in VPT [8].

Severe motor, sensory and psychiatric disorders can usually be diagnosed before age 2 years [9]. Cognitive and behavioral problems may be diagnosed later, at school age, and require longer-term follow-up [10]. All these findings strengthen the importance of early screening for children born preterm [9–10]. Follow-up, associated with perinatal network training schemes, may help in identifying developmental abnormalities and result in provision of early support systems, thus improving the functional outcome and quality of life of these patients and interaction with their parents.

Tremendous progress regarding life-support therapy and care for premature babies has been achieved in recent years [11]. To assess the impact of patient management and changes in the neurological outcome of infants at age 2 years, we conducted a retrospective observational study in our level 3 maternity hospital comparing VPT children born in 2000, 2005 and 2010.

## Methods

### Study population

This is a monocentric, descriptive and comparative cohort study. We included all VPT infants born before 33 weeks’ gestation and admitted to the neonatal intensive care unit (NICU) at Rouen University Hospital during 2000, 2005 and 2010. Rouen University Hospital is located in the French region of Haute-Normandie, with an average of 17000–18000 births per year. Exclusion criteria were a death before age 2 years Data at birth and at age two years were collected prospectively. We collected data from standardized questionnaires used in clinical practice in the unit [S1 Fig.]. These questionnaires have been used for several years and are based on neurodevelopemental scales validated in the literature. Follow-up was conducted between 22 and 26 months of chronological age by pediatricians from the neonatal unit of Rouen University Hospital or by pediatricians from peripheral hospitals where the children had been transferred before returning home. The parents of some children lost to follow-up were contacted by telephone by the same investigators. Data analysis was done retrospectively after collecting all the data. This study was approved by the Local Ethics Committee of Rouen University Hospital [S2 Fig.] and the French Data Protection Agency (Commission Nationale de l’Informatique et des Libertés). For participation, an information note was given to parents and written consent was obtained before each infant was included in the Perinatal network of Haute-Normandie [S3 and S4 Figs.].

### Neonatal data

In brief, we collected predefined data from the neonatal period, as already published: gestational age, sex, birth weight, antenatal corticosteroid therapy or administration of magnesium sulfate, chorioamniotitis (defined by the presence of at least two of the following signs: fetal tachycardia, maternal hyperthermia and/or biological inflammatory syndrome before delivery, stained or foul amniotic fluid, presence of germ in amniotic fluid), neonatal mortality, neonatal morbidities: chronic lung disease defined as oxygen dependence at 36 weeks’ gestational age, ulcerative necrotizing enterocolitis defined according to the Bell classification, nosocomial infection, materno fetal infection (probable in cases of clinical and/or laboratory signs of infection, certain in cases of positive blood culture or positive cerebrospinal fluid culture), treatment of ductus arteriosus, use of postnatal steroids, breastfeeding rates (breastfeeding alone or mixed feeding) at hospital discharge and extra uterine growth restriction (defined by a weight under the 10^th^ percentile according to WHO curves). Cranial ultrasonographic studies (cUS) were performed according to routine protocol to detect acquired cerebral lesions: subependymal/intraventricular hemorrhage (IVH) according to the Papile classification, or periventricular leukomalacia (PVL) defined as evidence of cystic lesions or persisting heterogenic hyperechogenicity at two cUS separated by at least an 8-day interval, in the periventricular area [11].

### Outcome data

At age 2 years, we collected the following data: weight, height and head circumference, and trophicity according to WHO (world health organization) curves. Motor development and cognitive function were assessed by routine score based on the Amiel-Tison and Denver developmental scales [9, 12]. Cerebral palsy was evaluated according to the European Cerebral Palsy Network definition [13]. Normal motor score was defined by walking before age 18 months, protected falls, ability to jump on two feet, run, climb stairs, stack at least two cubes, and crawl. Walking age was analyzed for each child. Presence of epilepsy was also investigated. Normal cognitive score was defined as the ability to build or make a puzzle, get dressed alone, engage in symbolic play, name an image, string two words together, point, and share with others. Normal psychosocial behavioral score was defined with items from the French version of the Strengths and Difficulties Questionnaire when the child is calm and does not require the intervention of a third person [14–15]. Normal sleeping score was defined as absence of sleeping disorders. Normal feeding score was defined as absence of feeding difficulties. For each score, in case of abnormal score, a severity scale was defined according to specific criteria: mild, moderate and severe impairment [S1 Table].

Comparisons between children examined at the consultation at two years with those whose the parents were interviewed by phone were done. We reported the number of infants lost to follow-up and compared them with infants followed.

### Statistical Analysis

For the qualitative variable, we used Chi2 test (or Fisher test if the numbers were too small) to compare outcomes between the three cohorts. For quantitative variables, we used ANOVA (ANalysis Of Variance) test followed by Newman and Keuls test if necessary. Differences between 2000, 2005 and 2010 were considered significant when p value was <5%. Statistical analysis was performed by NCSS software.

## Results

During neonatal period, we included 170 infants born in 2000, 173 born in 2005 and 193 in 2010 [Table 1, S2 Table].

**Table 1:** Flow chart of the study.

	2000	2005	2010
Neonatal period	170 patients	173 patients	193 patients
Death patient	13 patients (8%)	18 patients (10%)	14 patients (7%)
Lost to follow up	34 patients (22%)	11 patients (7%)	15 patients (8%)
Evaluation 2 years	123 patients	144 patients	164 patients

Table 2 reports the perinatal characteristics of the three cohorts [Table 2, S2 Table]. The neonatal mortality of children admitted was stable: 8% in 2000, 10% in 2005 and 7% in 2010. Most of the perinatal study variables showed no significant differences between the three cohorts. Antenatal magnesium sulfate was administered to 14% of women at risk of preterm birth and included in the PREMAG trial in 2000, none in 2005 and 61% of women in 2010 as we set up routine protocol in the obstetrical unit of our center from March 2010. Neonatal hospital morbidity remained roughly stable. The rate of intraventricular hemorrhage decreased but the decrease was only for grade 1 (77% in 2000, 51% in 2005 and 46% in 2010). The rate of periventricular leukomalacia has decreased since 2000, but the difference is not significant. We found five cavitary white matter lesions in 2000, one in 2005 and none in 2010. Use of post natal corticosteroids decreased for 11 years. Extra-uterine growth restriction increased in 2010 parallel to the increase in breastfeeding. No statistically significant differences in perinatal variables were found between patients lost to follow-up and those evaluated at age 2 years (data not shown).

**Table 2:** Perinatal characteristics of 2000, 2005 and 2010 cohorts.

	2000	2005	2010	P
Gender: male n/N (%)	102/170 (60)	90/173 (52)	107/193 (55)	0.33
Outborn n/N (%)	26/170 (15)	33/173 (19)	33/193 (17)	0.63
Birthweight (gram) mean ±SD	1328.2 ±429.9	1395.3 ±408.9	1332.3 ±391.7	0.23
Intra uterine growth restriction n/N (%)	23/170 (14)	22/172 (13)	18/193 (9)	0.34
Gestational age (weeks) mean ± SD	29.4 ±2.3	29.9 ±2.2	29.6 ±2.2	0.18
Antenatal corticosteroids n/N (%)	138/170 (81)	135/173 (78)	162/193 (84)	0.35
Magnesium sulfate n/N (%)	24/170 (14)	0/173 (0)	117/193 (61)	<0.001† #
Chorioamnionitis n/N (%)	32/170 (19)	29/173 (17)	17/193 (9)	0.015 †
Preeclampsia n/N (%)	27/170 (16)	25/173 (14)	39/193 (20)	0.31
Respiratory Distress Syndrome n/N (%)	78/170 (46)	93/173 (54)	90/193 (47)	0.23
Chronic Lung Disease n/N (%)	27/157 (17)	27/155 (17)	31/179 (17)	0.45
Necrotizing enterocolitis n/N (%)	7/169 (4)	4/173 (2)	7/193 (4)	0.63
Materno fetal infection n/N(%)	16/170 (9)	18/173 (10)	13/193 (7)	0.44
Nosocomial infection n/N (%)	51/170 (30)	51/173 (29)	52/193 (27)	0.79
Intraventricular hemorrhage n/N (%)	35/170 (21)	43/173 (25)	24/193 (12)	0.008 #
Periventricular leukomalacia n/N (%)	24/170 (14)	14/173 (8)	14/193 (7)	0.06
Postnatal corticosteroids n/N (%)	34/170 (20)	10/173 (6)	15/193 (8)	<0.001 † ■
Treatment of PDA n/N (%)	26/170 (15)	31/173 (18)	39/193 (20)	0.47
Parenteral nutrition (days) mean ± SD	19.8 ± 15.5	17.8 ± 21.9	14.6 ± 15.3	0.02 †
Breast-feeding n/N (%)	44/157 (28)	37/151 (25)	89/175 (51)	<0.001 † #
Extra uterine growth restriction n/N (%)	30/131 (23)	33/130 (25)	64/164 (39)	0.004† #

# Persistence difference between 2005 and 2010,

† Persistence difference between 2000 and 2010,

■ Persistence difference between 2000 and 2005SD: Standard deviation, PDA: patent ductus arteriosus

Evaluation at age 2 years was performed in 123 of 157 (78%) survivors of the 2000 cohort, 144 of 153 (94%) survivors of the 2005 cohort and 164 of 179 (92%) survivors of the 2010 cohort [Table 1, S2 Table]. Tables 3 and 4 report the main outcomes at age 2 years of the 2000, 2005 and 2010 cohorts [Table 3, Table 4, S2 Table]. Mean age at examination was 24.3 ± 1.8 months in 2000, 24.2 ± 2.3 months in 2005 and 24.9 ± 2.5 months in 2010. We did not find any difference between children followed in consultation and those contacted by phone (data not shown). Motor score improved over the 3 periods and cerebral palsy rate decreased significantly. The most common form of cerebral palsy was spastic diplegia:. 100% in 2010 (n/N = 1/1), 78% in 2005 (n/N = 7/9) and 80% (n/N = 12/15) in 2000. Other forms were hemiparesis. Normal cognitive score did not change significantly. A non-significant decrease in behavioral problems was observed. While extra-uterine growth restriction on discharge home during neonatal period increased over 11 years, there were no differences regarding anthropometric parameters at age 2 years.

**Table 3:** Outcomes at 2 years of age in the survivors of 2000, 2005 and 2010 cohorts.

	2000	2005	2010	P
**Normal motor score** n/N (%)	85/123 (69)	117/142 (82)	139/164 (85)	0.002 ■ †
Score 1 n (%)	85 (69)	117 (82)	139 (85)	
Score 2 n (%)	30 (24)	17 (12)	22 (13)	
Score 3 n (%)	7 (6)	4 (3)	2 (1)	
Score 4 n (%)	1 (1)	4 (3)	1 (1)	
**Cerebral palsy** n/N (%)	15/122 (12)	9/142 (6)	1/164 (1)	<0.001 †
**Walking age** (months) mean	15.8 ± 2.6	15.9 ± 3.4	16.1 ± 2.4	0.68
**Normal cognitive score** n/N (%)	80/122 (66)	89/141 (63)	113/163 (69)	0.52
Score 1 n (%)	80/122 (66)	89/141 (63)	113/163 (69)	
Score 2 n (%)	40/122 (33)	47/141 (33)	46/163 (28)	
Score 3 n (%)	2/122 (2)	4/141 (3)	4/163 (2)	
Score 4 n (%)	0/122 (0)	1/141 (1)	0/163 (0)	
**Epilepsy** n/N (%)	2/122 (2)	2/141 (1)	1/164 (1)	0.68
**Normal feeding score** n/N (%)	90/123 (73)	107/133 (80)	139/163 (85)	0.046 †
**Normal sleeping score** n/N (%)	99/123 (80)	109/135 (81)	134/164 (82)	0.93
**Normal behavior** n/N (%)	75/118 (64)	76/117 (65)	121/163 (74)	0.08

† Persistance difference between 2000 and 2010,

■ Persistance difference between 2000 and 2005

**Table 4:** Anthropometric data and parent’s level at 2 years of age.

	2005	2010	P
**Weight -2SD** n/N (%)	26/135 (19)	34/164 (21)	0.71
**Height -2SD** n/N (%)	17/132 (13)	22/163 (13)	0.69
**Head circumference—2SD** N (%)	16/124 (13)	26/163 (16)	0.42
**High school mother’s level** n/N (%)	53/78 (68)	82/147 (56)	0.06
**High school father’s level** n/N (%)	54/78 (69)	80/138 (58)	0.09

SD: Standard deviation

## Discussion

The three cohorts of VPT infants admitted to the NICU at Rouen University Hospital during three 5-year interval periods in 2000, 2005 and 2010 demonstrated the same characteristics in gestational age, birthweight and gender. Whereas we observed no or few changes in neonatal mortality and morbidity rates during the three periods, at age 2 years, we observed a significant improvement in neuromotor outcome, a dramatic decrease in cerebral palsy, a non-significant decrease in abnormal psychosocial behavior, and significant positive changes in feeding behavior. Nevertheless we were not able to observe differences in cognitive or sleeping outcomes.

Our survey is interesting as it allows assessment of the positive evolution of neurological outcome at age 2 years in VPT infants admitted to hospital in recent years in a representative region of France, with 17000 live births per year. Information collected from neonatal periods as well as neurological examinations at age 2 years were obtained with standardized questionnaire by the same homogeneous pediatric team. Information was collected by telephone interview in a small percentage of cases (3%). The latter has demonstrated accuracy regarding collection of information on child development and disabilities at age 2 years [16]. Our follow-up rates are close to those of other studies conducted by similar centers, but higher than population based-studies such as EPICURE or EPIPAGE [6, 17, 18]. However, our study has some limitations. The study is monocentric and the sample size is smaller than in population-based studies. Some parts of the standardized questionnaire are rough with low sensitivity in detecting minor abnormalities. Some children were lost to follow-up at two years. Even if there was no difference between neonatal brain lesions and major neonatal morbidities in the followed children and those lost to follow-up in our study, the literature found that infants lost to follow-up were more likely to have severe disabilities than those followed up [19]; and the highest rate (28%) of infants lost to follow-up was observed in children born in 2000, who displayed the highest rate of neuromotor deficiencies.

In our study, we found a significant and dramatic decrease in CP from 2000 to 2010, lowering from 12% to 1%. CP is the most common developmental disorder associated with lifelong motor impairment and disability. A decreased incidence of CP in VPT has already been observed in other studies and was usually attributed to a decrease in severe periventricular leukomalacia [4, 20]. Major cerebral lesions such as cavitary PVL and parenchymal hemorrhage were the most important predictors of CP [21]. In our study, rates of cavitary PVL were very low during the 3 years investigated (5 in 2000, 1 in 2005 and 0 in 2010) and did not change significantly. The sole difference in neonatal brain lesions concerned low-grade IVH, the significance of which remains to be clearly established. In a recent study, the neurodevelopmental outcomes of extremely low-gestational age infants with low-grade intraventricular hemorrhage were no different to those with normal cUS [22]. However this result is in disagreement with other studies, which observed a greater negative effect on CP rates or neurodevelopmental outcomes [6, 23]. Indeed CP can be observed in the absence of identified cerebral lesions. In addition, we identified multiple risks factors associated with CP in VPT infants: low gestational age, chorioamnionitis and neonatal sepsis, asphyxia at birth, birth defects, major but also less severe cerebral acquired lesions, post-natal corticosteroid therapy and intra- or extra- uterine growth retardation [7, 20, 24]. In our study, we assumed that improvement could be due to a decrease in rates of chorioamnionitis, which has been linked to CP, and also to several modifications in care for premature babies [25]. Use of post-natal corticosteroid therapy, which has been reported to adversely affect motor development when given to preterm infants less than 10–15 days after birth [26] has been significantly reduced in recent years in our neonatal department. Finally, in the department of obstetrics at Rouen University Hospital, antenatal magnesium sulfate has been administered to more than 90% of women at risk of preterm birth since March 2010. This could also explain the decrease observed in CP rates in our study between 2000 and 2010, as previously demonstrated by the significant neuroprotective effect of magnesium sulfate in randomized trials and the significant decrease in CP in meta-analysis [27–29]. Improvement in outcome between 2000 and 2005 is not related to magnesium sulphate (not used in 2005) but others parameters could explain decrease of CP rates: significant decrease of post natal corticosteroids therapy, non-significant decrease in necrotizing enterocolitis and periventricular leukomalacia rates.

Contrary to motor outcome, we did not find any differences in cognitive impairment at age 2 years during the 11 years of the study. The rates are around 30–35% which is higher than CP rates but similar to rates in other studies using more precise scale measurement [7, 30]. However, at age 2 years, they are likely underestimated as our measurement was rough. Cognitive impairment is difficult to diagnose early before it interferes with school learning from age 3 years. We are hopeful that it will also decrease in the future as it is positively influenced by breast feeding which increased, and a decrease in CP, to which it is frequently associated [21]. Even if non-significant, we observed a decrease in behavioral disorders and a significant increase in better feeding scores. These data are of importance as behavior significantly alters learning at school. Behavioral disorder frequency is estimated at 25–55% between age 3 and 8 years and in our survey concerned 25% to 35% of infants at age 2 years [31–33]. We hypothesized that several factors observed in ante- and neo-natal periods could be involved: decrease in post-natal corticosteroids, increase in breast feeding and use of antenatal magnesium sulfate [34].

In 2010, concomitantly with an increase in breast feeding, we were concerned by an increased rate in extra uterine growth restriction. Extra uterine growth restriction is a prevalent problem in premature infants [35] and associated with poor neurological development [36]. Outcome at age 2 years was more reassuring than at discharge from first admission to hospital: half the children with growth failure at discharge had caught up satisfactory weight growth at age 2 years. Extra uterine growth restriction rate at age 2 years was therefore similar in 2005 and 2010 and could not have been involved in a decline in neurological alterations as described in the literature.

Although our study observed several improvements in the overall outcomes of VPT infants at age 2 years, neonatal morbidity rates were only marginally modified. Neonatal morbidity may be associated with brain acquired hypoxic-ischemic/inflammatory lesions or other inflammatory diseases such as broncho-pulmonary dysplasia or enterocolitis, which are not the sole processes involved in the occurrence of neurodevelopmental disorders. Processes that guide neonatal maturation, motricity, cognition and behavior are also influenced by external factors, which alter environment. In our study we identified at least two contributing factors: nutritional factors with an increase in breast feeding and pharmacological factors with antenatal use of magnesium sulfate and a decrease in post-natal corticosteroid therapy.

## Conclusion

We observed significant improvements in neuromotor and feeding behavior outcomes at age 2 years in very preterm infants born in 2000, 2005 and 2010. However, we did not evidence any differences regarding cognitive impairment. Larger prospective population-based studies are needed to confirm these data. Extending our study to include infants at older age is important in order to diagnose later cognitive impairment, in particular attention and executive functioning disorders, and school-based learning difficulties, as well as to confirm the decreasing prevalence of neurodisabilities in VPT infants. Improving the neurological outcome of preterm babies remains a major challenge, requiring active research to develop neuroprotection strategies.

## Supporting Information

S1 Fig Questionnaires used in clinical practice in the unit for follow-up at 2 years. (DOC)

S2 Fig Study consent of the local ethics committee. (DOC)

S3 Fig Written parental consent (French version). (DOC)

S4 Fig Written parental consent (English version). (DOC)

S1 Table Neurodevelopmental score. (DOCX)

S2 Table Data table. (XLS)
