# Supplementary material for: Comparison in Outcomes at Two-Years of Age of Very Preterm Infants Born in 2000, 2005 and 2010
Source: PLoS One. 2015 Feb 6;10(2):e0114567. doi: 10.1371/journal.pone.0114567 (PMC4320065; doi:10.1371/journal.pone.0114567)
Supplement: S1 Fig — (DOC) [file pone.0114567.s001.doc]

Figure S1: Questionnaires used in clinical practice in the unit for follow-up at 2 years


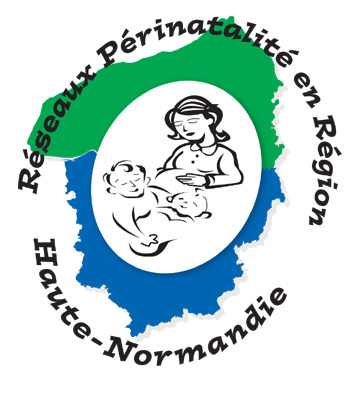


FOLLOW-UP at 2 years (22-26 months)

**IDENTIFICATION**

**|__|__|__| |__|__| |__|__|__|**

Name First name Inclusion number

**(Do not fill)**

| ***Coordination :***  **Catherine LEVEQUE**  Department of Pediatrics Neonatal and Resuscitation  Mother and Child Pavillon  Charles Nicolle Hospital  C.H.U. de Rouen  1 rue de Germont  76031 ROUEN Cedex  Phone : 02.32.88.01.24  Fax : 02.32.88.88.23  E-mail : [Catherine.Leveque@chu-rouen.fr](mailto:Catherine.Leveque@chu-rouen.fr) |
| --- |

June 2007 version

**NOTES FOR THE USE OF THE QUESTIONNAIRE**

- Fill each item
- For DATES items:

Complete boxes  D  D   M  M   Y  Y  (day / month / year)

- For items in coded numbers:

NO = 1

YES = 2

DNK (does not know) = 9 or 99, if the answer is unknown

-- = 0, if no answer

- For SCORE items:

- Copy in the box the number that best fits the situation encountered (the higher the score, the higher the severity).

- It is possible that some items will not recover for the selected score. The majority of items should be found in the selected score.

- It is possible to remove items not found in the selected score

- For TEXT items:

Please specify in full text on the corresponding dotted lines.

- For items with the mention "possible association":

You can check multiple boxes.

- For items on the "GROWTH":

Curves are available on pages 3-4 for boys ♂

and pages 5-6 for girls ♀.


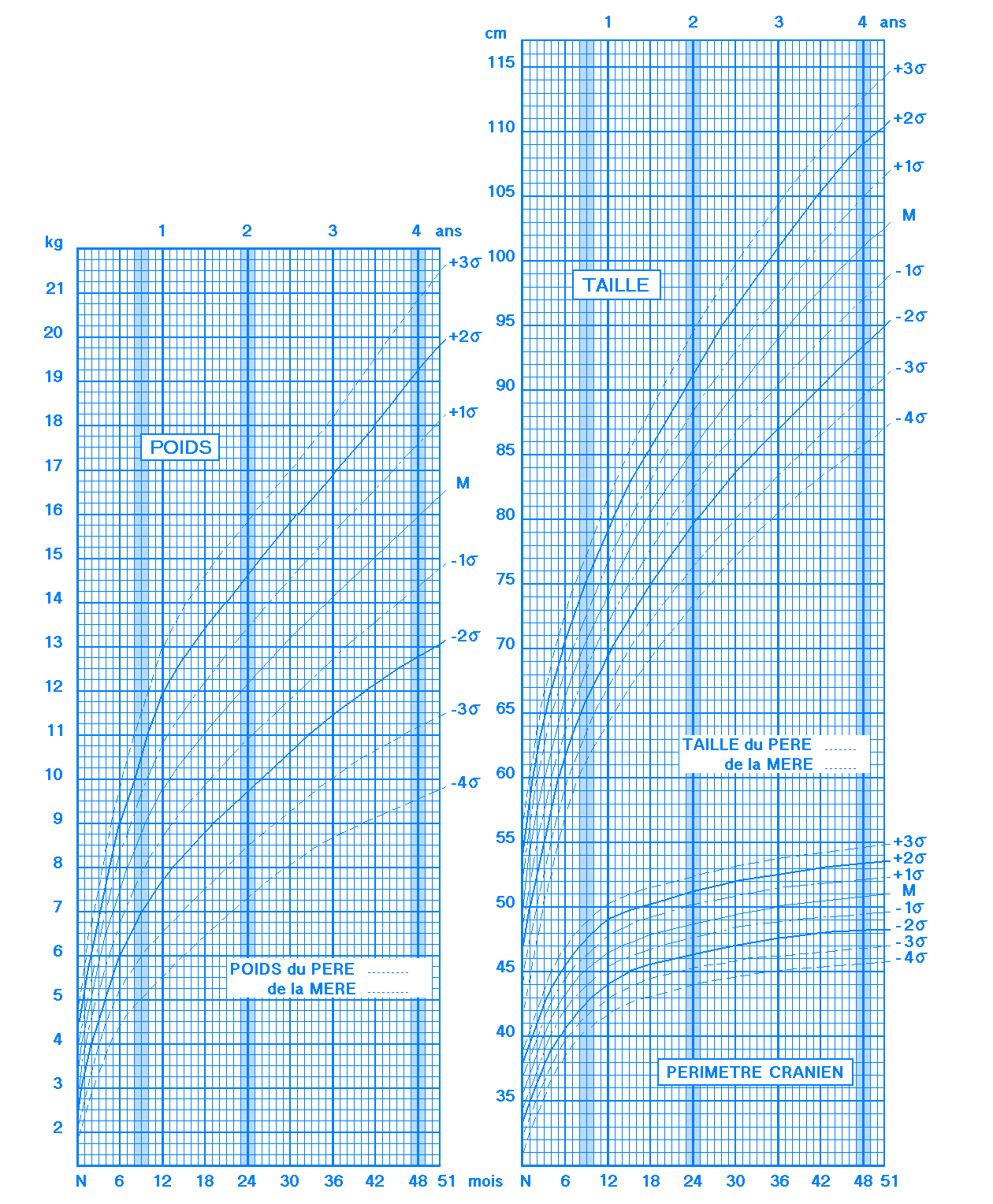
Somatic growth of boys from birth to 4 years 3 months (Sempé and Pedron)

♂

**WEIGHT**

**SIZE**

**year**

Head circumference

**year**

**month**

**WEIGHT of Father …………..**

**Maternal WEIGHT ……………**

**SIZE of father …………..**

**of mother …………**


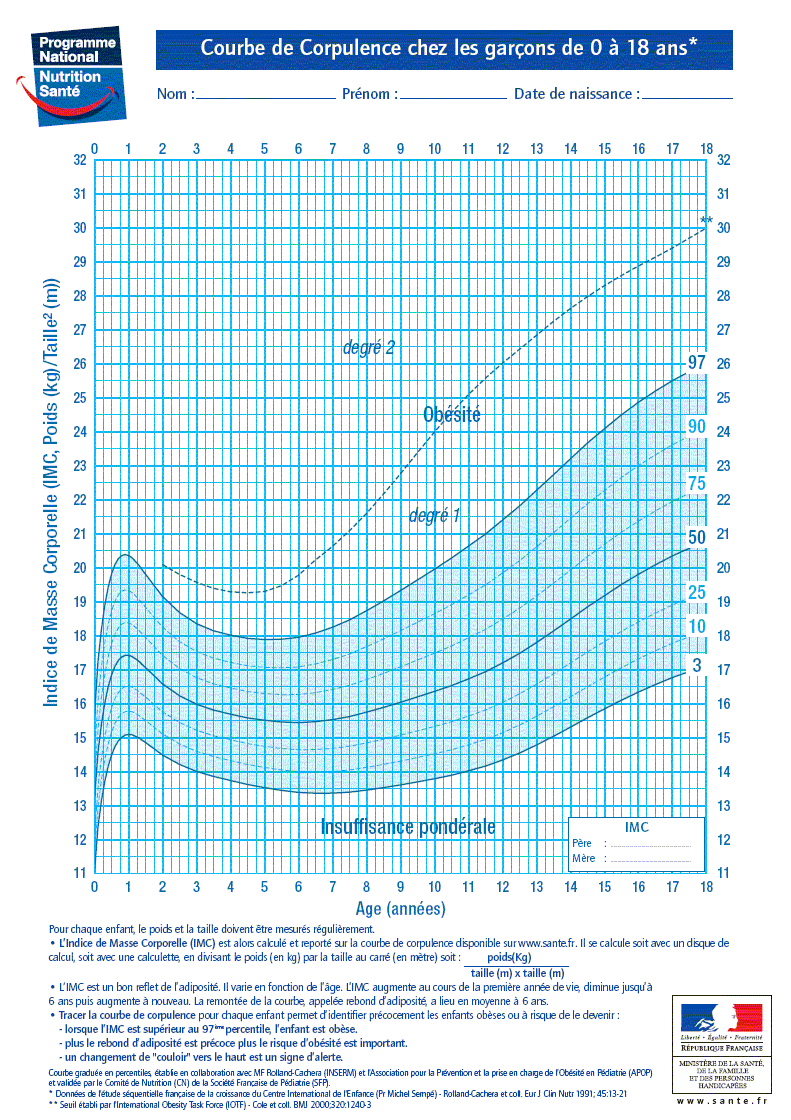


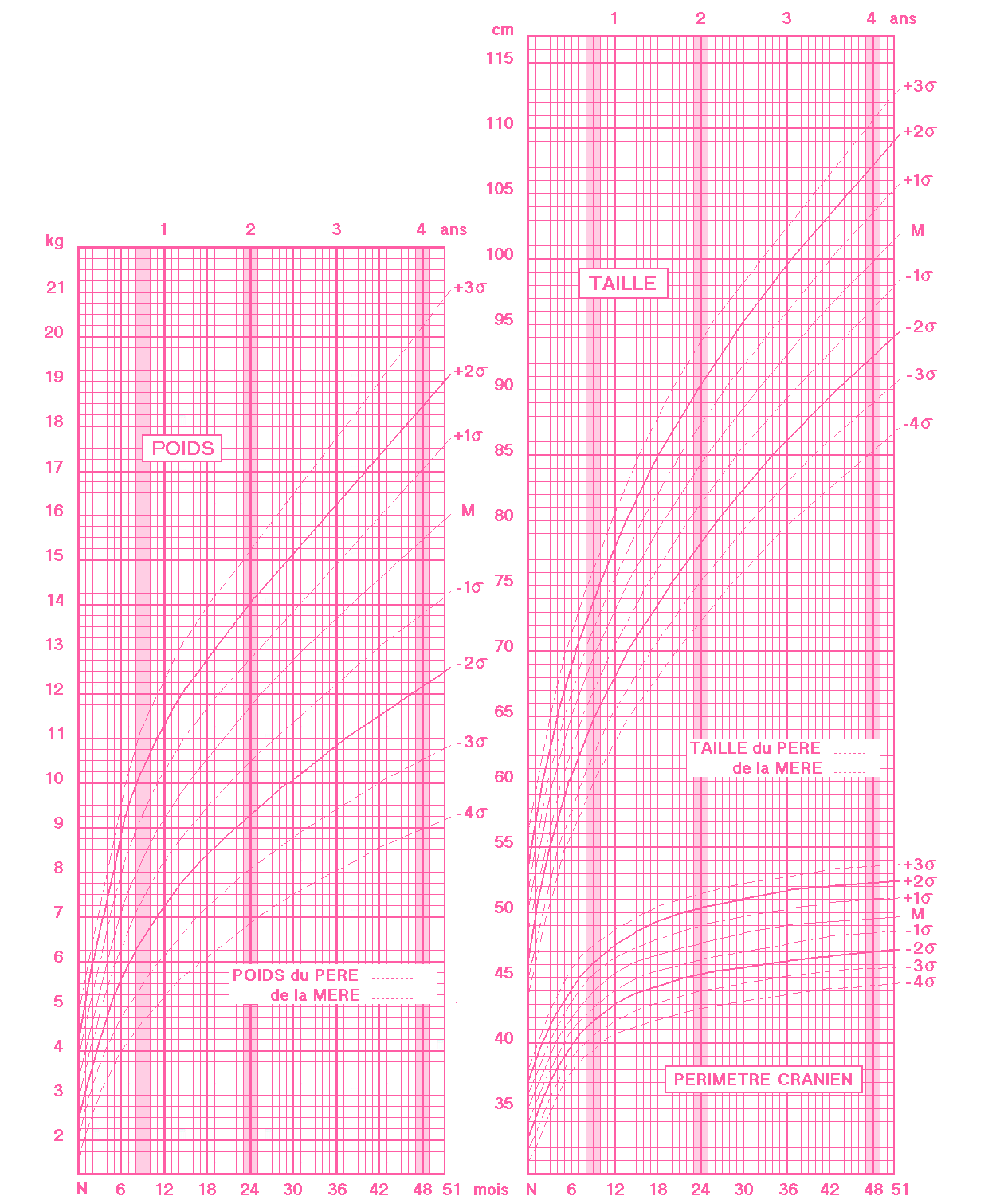
Croissance somatique des filles de la naissance à 4 ans 3 mois (Sempé et Pédron)

**♀**

**WEIGHT**

**SIZE**

**year**

**year**

**month**

**WEIGHT of Father ………**

**Maternal WEIGHT ……….**

**SIZE of father …………..**

**of mother …………**

Head circumference


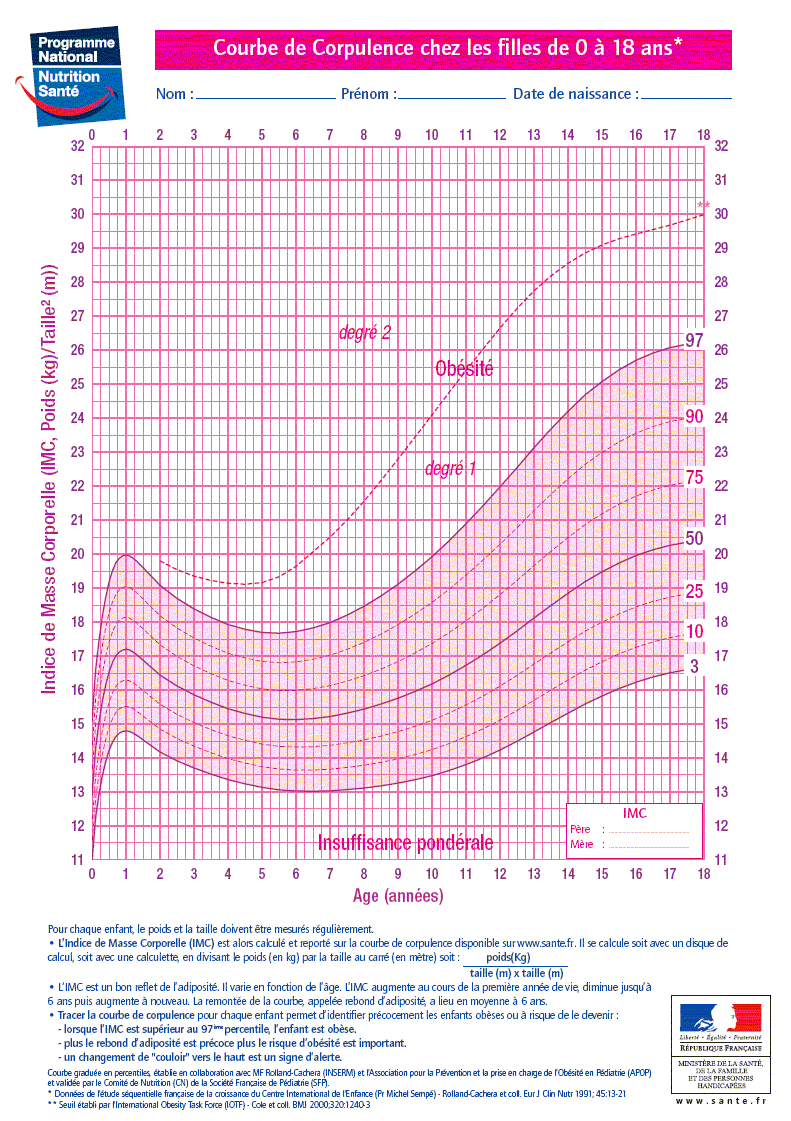


# MONITORING TO 2 YEARS BETWEEN 22 AND 26 MONTHS (actual age)

# **Filling powered by:** ___________________________

# **Date filling:** ____ ____ ____

**Medical examination**

# **Doctor's Name:** ___________________________

# **Speciality** (Practitioner=1, Paediatrician=2, Other specialist=3, DNK=9, --=0): __

# **Location Monitoring** (Private practice=1, Health center consultation=2, Hospital consultation=3, Other=4, DNK=9, --=0): __

# **Others professionals who participated in follow-up** (possible association):

Neonatologist and paediatrician in hospital ❑ 1

Private Paediatrician ❑ 2

General Practitioner ❑ 3

Doctor in Health Center ❑ 4

Doctor in specialized center ❑ 5

Others professionals ❑ 6

DNK ❑ 9

If others professionals 🗹 (underline): Surgeon, Physiotherapists (respiratory and/or motor), Ophthalmologist, Speech Therapist, Orthoptist, Otho-Nose-laringologiste, Child Psychiatrist, Psychologist, Psychomotricien, Others (please specify):

# **Reasons of specialized monitoring** (possible association):

Premature infant whose term birth is ≤ 34 weeks ❑ 1

Child whose weight is < 3rd percentile and whose term birth is ≥ 35 weeks ❑ 2

Child with suspected fetal alcohol syndrome ❑ 3

Children with cerebral abnormality diagnosed during antenatal period ❑ 4

Another serious cases to the term birth requiring follow ❑ 5

DNK ❑ 9

If brain abnormalities 🗹 (underline): Agenesis of the corpus callosum, Agenesis of the septum, Cerebellar vermis agenesis, Ventricular asymmetry, Ventricular dilatation, Septal dysplasia, Hydranencephaly, Cerebellar hypoplasia, Hypoplasia of the corpus callosum, Arachnoid cyst, Cyst cerebellar, Lissencephaly, Microcephaly, Porencephaly, Schizencephaly, Others (please specify):

If others serious cases 🗹 (underline): Cerebral Vascular Accident, Convulsions neonatal, Hypoxic-ischemia, Severe jaundice, Heart disease cyanogen, Others (please specify):

**Child**

1. **Place of birth**: ___________________________
2. **Date of Birth**: ____ ____ ____
3. **Date of consultation**: ____ ____ ____
4. **Real Age of child at the consultation** (months + days): ____ + __
5. **Sex** (Male ♂=1, Female ♀=2, Indeterminate=3, DNK=9, --=0): __

**GROWTH** (measures in consultation) curves boys ♂ (p3-4) and girls ♀ (p5-6).

1. **Weight during the consultation** (kg , g): ____ , ______

Correspondence in standard deviation (SD): + / - |__|,|__|

1. **Size during the consultation** (cm): ____ , __

Correspondence in standard deviation (SD): + / - |__|,|__|

1. **Body Mass Index** (BMI = kg/m^2^): ____ , __

Correspondence in percentile (%): |__|__|

1. **Cranial size** (cm): ____ , __

Correspondence in standard deviation (SD): + / - |__|,|__|

1. **Size of mother** (m): __ , ____
2. **Size of father** (m): __ , ____

**Cardio-respiratory function**

1. **Systolic Blood Pressure** (mm Hg): ______
2. **Diastolic Blood Pressure** (mm Hg): ______
3. **Respiratory score** (between 1 and 2 years): __

| 1 | No problem |
| --- | --- |
| 2 | Bronchitis / bronchiolitis occasional and mild |
| 3 | Bronchitis / bronchiolitis repeated (≥ 3)  Bronchitis / bronchiolitis requiring hospitalization  Frequent wheezing  Prolonged inhaled treatment (please specify length):  Prolonged physiotherapy (please specify length): |
| 4 | Oxygen treatment  Tracheotomy |
| 5 | Signs of struggle (draft) at 2 years |
| 9 | DNK |
| Details (duration of treatment, etc ...): | |

1. **Hospitalization for bronchiolitis between 1 and 2 years**

(No=1, Yes=2, DNK=9, --=0): __

If yes, please specify: Number of bronchiolitis with hospitalization: ____

Number of days on oxygen (O_2_): ____

Number of days on mechanical ventilation: ____

1. **Biological sampling between 1 and 2 years** (No=1, Yes=2, DNK=9, --=0): __

If yes, result: Respiratory Syncytial Virus (RSV) - ❑ 1

Respiratory Syncytial Virus (RSV) + ❑ 2

DNK ❑ 9

1. **Treatment anti-RSV between 1 and 2 years** (No=1, Yes=2, DNK=9, --=0): __
2. **Functional respiratory tests (PFT) between 1 and 2 years**

(No=1, Yes=2, DNK=9, --=0): __

If yes, result: Normal ❑ 1

Restrictive syndrome ❑ 2

Obstructive syndrome ❑ 3

DNK ❑ 9

1. **Passive smoking** (No=1, Yes=2, DNK=9, --=0): __

If yes, result: Father ❑ 1

Mother ❑ 2

Father + Mother ❑ 3

DNK ❑ 9

1. **Atopic field family** (No=1, Yes=2, DNK=9, --=0): __

If yes, please specify:

**Neurosensory function**

1. **Motor score** (22-26 months): __

| 1 | Run, up the stairs, jump on two feet  Protected falls when he run  Start scribbling himself or invitation  Stacks at least two cubes |
| --- | --- |
| 2 | Walking after 18 months  Unprotected falls  Direct approach to the subject, capture crumbs |
| 3 | Abnormal walking or assisted walking (diplegia)  Use very preferably a hand  Approach very rough of the object  No precision grip |
| 4 | No walking even assisted  Like hardly sitting (quadriplegia)  No approach to the object  Pathological grip |
| 9 | DNK |
| Details (hypertonia, hypotonia, lower and / or upper limbs, etc ...): | |

1. **Age of walking** (months): ____
2. **Cerebral Palsy (CP)** (No=1, Yes=2, DNK=9, --=0): __
3. **Anti-epileptic treatment between 1 and 2 years** (No=1, Yes=2, DNK=9, --=0): __
4. **Brain imaging between 1 and 2 years** (No=1, Yes=2, DNK=9, --=0): __

If yes, result: Normal ❑ 1

(IRM) Pathological ❑ 2

DNK ❑ 9

If result is pathological, please specify:

1. **Cognitive score** (22-26 months): __

| 1 | Flush with reflection and application (puzzle)  Wants to dress himself  Pretends to call (with or without phone)  Appoints picture, assembles two words  Identification game with her doll or his teddy  Seeks to share attention and exchange |
| --- | --- |
| 2 | Designates the object or animal on picture without naming  Means the parts of his body  More labile attention, get tired quickly  random recesses  No identification game  Use single words, agrees to repeat |
| 3 | No preference towards an object or activity  Only move empty and throws, not back in the box  Gibberish understandable without words  Do not know how to express his desire through gesture or attitude |
| 4 | Very stereotyped activities  Do not point the finger, do not follow the gaze  Production of stereotyped sounds  Self or hetero aggressive |
| 9 | DNK |
| Details: | |

1. **Visual score** (based on interview and medical examination): __

| 1 | Good visual tracking (180°) with eyes parallelism |
| --- | --- |
| 2 | Strabismus with good fixation of each eye and good continuation  Myopia |
| 3 | Strabismus with poor fixation of one eye  Disturbed continuation |
| 4 | Capping look  No ocular pursuit |
| 9 | DNK |
| Details: | |

1. **Medical examination by an ophthalmologist between 1 and 2 years**

(No=1, Yes=2, DNK=9, --=0): __

If yes, result: Normal ❑ 1

Pathological ❑ 2

DNK ❑ 9

If result is pathological, please specify:

1. **Glasses between 1 and 2 years** (No=1, Yes=2, DNK=9, --=0): __
2. **Auditory score** (based on interview and medical examination): __

| 1 | Appropriate responses to voice and noise |
| --- | --- |
| 2 | Responses with suspicious orientation |
| 3 | No reply |
| 9 | DNK |
| Details:: | |

1. **Medical examination by an specialist doctor between 1 and 2 years**

(No=1, Yes=2, DNK=9, --=0): __

If yes, result: Normal ❑ 1

Pathological ❑ 2

DNK ❑ 9

If result is pathological, please specify:

1. **Tympanostomy aerators** **between 1 and 2 years** (No=1, Yes=2, DNK=9, --=0): __
2. **Adenoidectomy between 1 and 2 years** (No=1, Yes=2, DNK=9, --=0): __

**Behavioural development**

1. **Sleeping score** (between 1 and 2 years): __

| 1 | No problem |
| --- | --- |
| 2 | Small problems easily solved |
| 3 | Moderate difficulties requiring special involvement of parents to manage the problem |
| 4 | Major difficulties: daily concern to tolerable limits for parents |
| 9 | DNK |
| Details (sleep disorders: difficulty falling asleep, nocturnal awakenings, nightmares, etc ...): | |

1. **Feeding score** (between 1 and 2 years): __

| 1 | No problem |
| --- | --- |
| 2 | Small problems easily solved |
| 3 | Moderate difficulties requiring special involvement of parents to manage the problem |
| 4 | Major difficulties: daily concern to tolerable limits for parents |
| 9 | DNK |
| Details (eating disorders: appetite, texture, types of food, anorexia, food allergy, etc …): | |

1. **Psychosocial score (behavior / social skills)** (22-26 month) : __

| 1 | No problem |
| --- | --- |
| 2 | Demanding child, calming easily |
| 3 | Passive or restless child, requiring parental involvement |
| 4 | Restless child,, consolable with difficulty or extremely passive |
| 9 | DNK |
| Details (behavioural disorders: hyperactivity, elective difficulties with the mother and / or father, maladjustments in another environment, etc ...): | |

**Health problems between 1 and 2 years**

1. **Readmissions** (No=1, Yes=2, DNK=9, --=0): __

If yes, number of readmissions: ____

**1** Service (Medicine=1, Surgery=2, DNK=9, --=0): __

Duration (days): ____

Cause:

**2** Service (Medicine=1, Surgery=2, DNK=9, --=0): __

Duration (days): ____

Cause:

**3** Service (Medicine=1, Surgery=2, DNK=9, --=0): __

Duration (days): ____

Cause:

**4** Service (Medicine=1, Surgery=2, DNK=9, --=0): __

Duration (days): ____

Cause:

**5** Service (Medicine=1, Surgery=2, DNK=9, --=0): __

Duration (days): ____

Cause:

1. **Other diseases between 1 and 2 years** (No=1, Yes=2, DNK=9, --=0): __

If yes, diseases: Nervous system (Spina, CP, Other *) ❑ 1

(possible association) Cardiovascular system (Congenital Heart Disease, Other *) ❑ 2

Respiratory system (Asthma, Cystic Fibrosis, Other *) ❑ 3

Digestive system (GERD, Other *) ❑ 4

Metabolic disease * ❑ 5

Endocrine disease * ❑ 6

Genital and urinary system (urinary and genital malformations, Other *) ❑ 7

Osteo-articular and muscular apparatus (dislocation of the hip, Other *) ❑ 8

Skin disease (Eczema, Other *) ❑ 9

Hematological diseases (hemoglobin disease, Other *) ❑ 10

Cleft lip and palate ❑ 11

Malformative syndrome * ❑ 12

Chromosomal aberrations (Trisomy 21, Other *) ❑ 13

Other disease detected * ❑ 14

DNK ❑ 99

* Details:

**Various**

1. **Number of children living at home**: ____
2. **Babysitting between 1 and 2 years (day AND night)** (possible association):

Parents ❑ 1

Family assistant ❑ 2

Nursery ❑ 3

Other, please specify: ❑ 4

DNK ❑ 9

1. **Modes of care between 1 and 2 years** (possible association):

Collective nursery ❑ 1

parental nursery ❑ 2

Childcare ❑ 3

Mixed reception ❑ 4

Home by a third party (eg family member) ❑ 5

Childminder (family nursery) ❑ 6

Childminder (independent) ❑ 7

Other, please specify: ❑ 8

DNK ❑ 9

If 1, 2, 3 or 4, Date of entry into collectivity ____ ____ ____

please specify: Age of entry into collectivity (months) ____

1. **Study maternal level**: __

| 1 | Primary school |
| --- | --- |
| 2 | Secondary |
| 3 | Bachelor level |
| 4 | Higher Education |
| 9 | DNK |

1. **Mother poorly controlled French (written and/or spoken)**

(No=1, Yes=2, DNK=9, --=0): __

1. **Study paternal level**: __

| 1 | Primary school |
| --- | --- |
| 2 | Secondary |
| 3 | Bachelor level |
| 4 | Higher Education |
| 9 | DNK |

1. **Father poorly controlled French (written and/or spoken)**

(No=1, Yes=2, DNK=9, --=0): __

**Vaccinations between 1 and 2 years** (possible association):

1. **Diphtheria Tetanus Polio** 1^e^ dose ❑ 1 2^e^ dose ❑ 2 3^e^ dose ❑ 3 Rappel ❑ 4 DNK ❑ 9
2. **Pertussis** 1^e^ dose ❑ 1 2^e^ dose ❑ 2 3^e^ dose ❑ 3 Rappel ❑ 4 DNK ❑ 9
3. **Hæmophilus influenza** 1^e^ dose ❑ 1 2^e^ dose ❑ 2 3^e^ dose ❑ 3 Rappel ❑ 4 DNK ❑ 9
4. **Hepatitis B virus** 1^e^ dose ❑ 1 2^e^ dose ❑ 2 3^e^ dose ❑ 3 Rappel ❑ 4 DNK ❑ 9
5. **Pneumococcus** 1^e^ dose ❑ 1 2^e^ dose ❑ 2 3^e^ dose ❑ 3 Rappel ❑ 4 DNK ❑ 9
6. **Rubella Oreillon Measles** 1 dose ❑ 1 2 doses ❑ 2
7. **BCG** (No=1, Yes=2, DNK=9, --=0) : __
8. **Other vaccines, please specify** (grippe, etc…):

**Overall assessment of the child** (health, neuropsychological development, behaviour, etc ...)

1. **Score established by parents at to 2 years:** __

| 1 | No problem |
| --- | --- |
| 2 | Occasional problems easily solved |
| 3 | Repetitive problems or unresolved |
| 4 | Troublesome problems of daily living or requiring multiple hospitalizations |
| 9 | DNK |
| Details: | |

1. **Score established by the doctor at to 2 years**: __

| 1 | No problem |
| --- | --- |
| 2 | Occasional problems easily solved |
| 3 | Repetitive problems or unresolved |
| 4 | Troublesome problems of daily living or requiring multiple hospitalizations |
| 9 | DNK |
| Details: | |

1. **Conclusions, clarifications and comments** (specific family situations: investment, death, divorce, incarceration, etc ...):...

THANK YOU
