# Supplementary material for: Comparison in Outcomes at Two-Years of Age of Very Preterm Infants Born in 2000, 2005 and 2010
Source: PLoS One. 2015 Feb 6;10(2):e0114567. doi: 10.1371/journal.pone.0114567 (PMC4320065; doi:10.1371/journal.pone.0114567)

Figure S2 : Study consent of the local ethics committee

Comité d'Ethique de la Recherche non-interventionnelle

CHU de Rouen

***Président : Pr Luc-Marie JOLY***

***Vice Président : Pr Jean-Jacques TUECH***

***Membres :***

***Pr Loïc FAVENNEC***

***Pr Isabelle MARIE***

***Dr Didier PINQUIER***

***Dr Horace ROMAN***

***Dr Eric VERIN***

***Rouen le***  15 - 7 - 2014

Le comité d'éthique de la recherche du CHU de Rouen a examiné le courrier et le protocole (E2014-19) du Dr Stéphane MARRET et de son équipe intitulé **:** " **Comparison in outcomes at two-years of age of very preterm infants born in 2000, 2005 and 2010.** "

Ce protocole propose de rapporter les résultats d'une étude non interventionelle, ne comportant pas de procédure supplémentaire de diagnostic ou de surveillance conformément à l'article R 1121-2 du CSP.

*Ce protocole ne pose pas de problème éthique et se trouve en conformité avec la loi française sur la recherche non-interventionnelle.*

*Le comité donne un avis favorable.*

***Professeur Luc-Marie JOLY***

***Président***


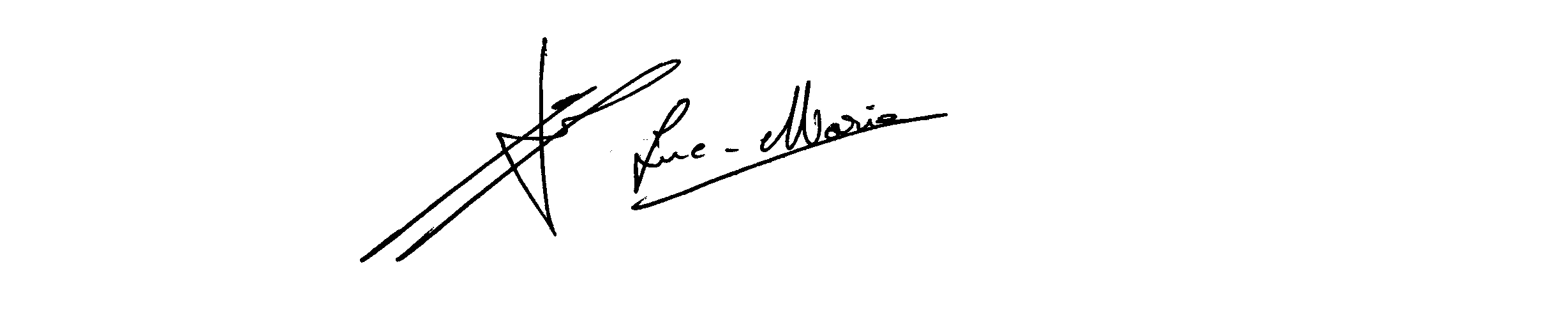

Supplement: S2 Fig — (DOC) [file pone.0114567.s002.doc]
