# Supplementary material for: Comparison in Outcomes at Two-Years of Age of Very Preterm Infants Born in 2000, 2005 and 2010
Source: PLoS One. 2015 Feb 6;10(2):e0114567. doi: 10.1371/journal.pone.0114567 (PMC4320065; doi:10.1371/journal.pone.0114567)
Supplement: S3 Fig — (DOC) [file pone.0114567.s003.doc]

**Figure S3 : Written parental consent (French version)**

**Evaluation du développement neurologique des enfants prématurés nés en 2010 hospitalisés au CHU de ROUEN**

Evaluation du devenir neurologique des enfants prématurés nés en 2010 hospitalisés au CHU de Rouen.

**NOTE INFORMATION ET CONSENTEMENT**

Madame, Monsieur ;

Dans le cadre de sa prématurité, votre enfant est régulièrement suivi en consultation spécialisée de pédiatrie. Afin d’améliorer la prise en charge des enfants nés prématurés, il est essentiel de connaitre leur devenir notamment sur leur développement neurologique.

Pour cela, dans le cadre d’un mémoire de DES de pédiatrie, nous menons une étude afin d’évaluer le devenir à 2 ans des enfants nés en 2010.

Nous souhaiterions effectuer ce suivi pour votre enfant et recueillir des informations auprès de son pédiatre sur son développement neuro-psychomoteur.

L’inclusion de votre enfant nécessite votre consentement écrit. Vous avez la possibilité de refuser de participer à cette étude. Si vous acceptez nous contacterons son médecin et accéderons à son dossier médical mais cela ne nécessitera aucune consultation supplémentaire.

En cas d’accord de votre part, merci de bien vouloir nous renvoyer le consentement ci-joint signé.

J’autorise l’accès aux informations médicales de la consultation de suivi à 2 ans de mon enfant I__________________________________I afin que celles-ci soient utilisées à visée épidémiologique.

Je déclare avoir été informé(e) de l’intérêt et des modalités de l’étude par le pédiatre DR I______________I. J’ai reçu une information claire et appropriée sur cette étude.

A I_______________________ I , le I_____________________I

Signature :

Pr Stéphane MARRET Lénaïg ABILY-DONVAL

Directeur de mémoire Interne
