# Supplementary material for: Comparison in Outcomes at Two-Years of Age of Very Preterm Infants Born in 2000, 2005 and 2010
Source: PLoS One. 2015 Feb 6;10(2):e0114567. doi: 10.1371/journal.pone.0114567 (PMC4320065; doi:10.1371/journal.pone.0114567)
Supplement: S4 Fig — (DOC) [file pone.0114567.s004.doc]

**Figure S4 : Written parental consent (English version)**

**Neurodevelopmental outcome at two-years of age of very preterm infants born in 2000, 2005 and 2010 in Rouen University Hospital**

Evaluation du devenir neurologique des enfants prématurés nés en 2010 hospitalisés au CHU de Rouen.

**INFORMATION AND CONSENT**

Madame, Monsieur ;

Due to preterm birth, your child is followed by a pediatrician from a neonatal unit. In order to enhance management of pre term infants, we need to evaluate their neurological outcome.

For a student working, we conduct a study to know neurological outcome of children.

We would like to follow up your child and to collect some information about his neurodevelopmental outcome.

Before inclusion of your child, we need to have your written consent. You can refuse to participate. If you accept, we’ll contact his pediatrician to collect information in his medical record. It will not require any additional consultation.

If agreed by you, thank you kindly return the attached consent signed.

I authorize access to medical information of my child I__________________________________I so that they are used for epidemiological purposes.

I declare I have been informed of the interest and the terms of the study by the pediatrician Dr. I______________I. I received clear and appropriate information on this study.

In I_______________________ I , the I_____________________I

Signature :

Pr Stéphane MARRET Lénaïg ABILY-DONVAL

Supervisor Medical student
