# Supplementary material for: Comparison in Outcomes at Two-Years of Age of Very Preterm Infants Born in 2000, 2005 and 2010
Source: PLoS One. 2015 Feb 6;10(2):e0114567. doi: 10.1371/journal.pone.0114567 (PMC4320065; doi:10.1371/journal.pone.0114567)
Supplement: S1 Table — (DOCX) [file pone.0114567.s005.docx]

**Table S1 : Neurodevelopmental score**

|  | **1: Normal, no disability.** | **2: Mild disability.** | **3: Moderate disability.** | **4: Severe disability.** |
| --- | --- | --- | --- | --- |
| **Motor score** | -Walking age before 18 months.  -Protected falls.  -Can run up the stairs, jump on two feet.  -Starts to scrawl.  -Stacks 2 cubes. | -Walking age after 18 months.  -Non protected falls.  -Direct approach to the subject. | -Abnormal walking, walking with assistance. (diplegia)  -Preferential use of a hand.  -No precision grip.  -Approximate approach of objects. | -No walking, even assisted.  -Quadriplegia.  -No approach of objects.  -Pathological grip. |
| **Cognitive score** | -Builds puzzles with reflection.  -Wants to dress himself.  -Identification game.  -Pretends to call.  -Names an image.  -Associates 2 words.  -Wants to share attention. | -Refers to the object on an image without naming it.  -Knows the parts of his body.  -Labile attention.  -Random embedding.  -No identification games.  -Uses isolated words, repeats words. | -No preference towards an object or activity.  -Empties and throws objects, does not call in the box.  -No understandable words.  -Does not know how to express his desire through gesture. | -Stereotyped activities.  -No pointing, no eye tracking.  -Vocalization, no language.  -Auto or hetero aggressive. |
| **Feeding Score** | -No problem. | -Minor problems easily resolved. | -Average difficulty requiring parental involvement in managing the problem. | -Significant difficulties in daily concerns with tolerable limits. |
| **Behavioral score** | -No problem. | -Child requiring calming, easily performed. | - Restless child requiring intervention from environment. | -Restless child, hardly consolable. |
| **Sleeping score** | -No problem. | -Minor problems easily resolved. | -Average difficulty requiring parental involvement managing the problem. | -Significant difficulties in daily concerns, tolerable limits. |
